# Extending voxel-based lesion mapping to neurosurgical oncology: applications in intracranial meningiomas

**DOI:** 10.3389/fonc.2026.1736621

**Published:** 2026-03-27

**Authors:** Aldo Spolaore, Sophie Wang, Kathrin Machetanz, Marcos Tatagiba, Georgios Naros

**Affiliations:** Department of Neurosurgery and Neurotechnology, Eberhard Karls University, Tuebingen, Germany

**Keywords:** histological subtype, meningioma, radiogenomics, spatial distribution, voxel-based lesion symptom mapping, dural attachement zone

## Abstract

**Background:**

Voxel-based lesion symptom mapping (VLSM) is a powerful neuroimaging technique for linking lesion location to clinical variables, widely used in stroke research. However, its methodological application in neuro-oncology, particularly for extra-axial tumors such as meningiomas, remains underexplored. This study aims to establish a methodological framework for extending VLSM to neurosurgical oncology by adapting it to the specific anatomical and biological features of meningiomas.

**Methods:**

In this retrospective single-center study, we analyzed preoperative MRI data from 350 patients who underwent surgical resection of intracranial meningiomas. Tumors were semi-automatically segmented, normalized to MNI space, and mapped to identify the dural attachment zone (DAZ). VLSM was performed using univariable linear regression and corrected for multiple comparisons to assess spatial correlations with tumor histology, preoperative symptoms, surgical outcomes, and postoperative deficits.

**Results:**

Meningiomas clustered predominantly along the anterior and middle skull base, falcine region, and sphenoid wing. WHO grade 2 meningiomas showed significant spatial clustering at the cerebral convexity and frontal base. Tumors involving the perirolandic area were associated with higher rates of pre- and postoperative motor deficits. Incomplete resections were more frequent in meningiomas of the medial sphenoid wing. VLSM results based on DAZ improved anatomical specificity and reduced volume-related bias compared to analyses using total tumor volume.

**Conclusion:**

VLSM offers a robust and anatomically precise framework for studying spatial patterns in meningioma biology and outcomes. Focusing on the DAZ enhances interpretability by reducing volume bias. This approach enables refined risk stratification and supports multicenter collaboration for developing spatially informed, individualized treatment strategies in neurosurgical oncology.

## Introduction

Voxel-based lesion–symptom mapping (VLSM) relates lesion location to clinical phenotypes using voxel-wise statistics in a common stereotactic space. Lesion masks from multiple patients are overlaid, and for each voxel an association test is performed between voxel involvement and the endpoint of interest, yielding statistical maps of regions linked to the phenotype. Although VLSM is well established in stroke research, applying it to extra-axial tumors requires careful handling of tumor growth and volume effects. VLSM has emerged as a transformative method for elucidating relationships between brain lesions and clinical outcomes (1, 2). Over the past two decades, VLSM has significantly advanced our understanding of the neural substrates of cognition, sensory processing, and motor control, particularly within neurological research on stroke, traumatic brain injury, and neurodegenerative disorders (1, 2). Despite its methodological maturity and widespread adoption in neurology, the application of VLSM in neurosurgical and neuro-oncological research remains limited. Only few VLSM studies have linked glioma location to molecular characteristics (3), preoperative seizures (4) and functional outcome (5).

Meningiomas, the most common primary intracranial tumor in adults (6), is in particular suitable for VLSM based analysis. Their well-delineated extra-axial anatomical boundaries facilitate precise lesion segmentation (7). Unlike intra-axial tumors, meningiomas primarily displace rather than infiltrate adjacent brain structures, minimizing distortion of deep white matter structures and thus enabling more accurate non-linear registration to standardized brain templates (8, 9). Until now, most meningioma location studies have relied on manually defined regions of interest (ROIs)—for example convexity, parasagittal, or sphenoid-wing tumors—that vary in exact boundary definitions across centers and effectively bucket continuous spatial variation into a handful of discrete categories. Only a few prior studies have employed VLSM to characterize the spatial distribution of intracranial meningiomas (10, 11), and even fewer have integrated histopathological or genetic data into the analysis (12). In a landmark study, Patel et al. (12) applied voxel-based lesion mapping to a large cohort of 881 patients, creating an atlas of the intracranial distribution of meningiomas. Their findings demonstrated that benign meningiomas were predominantly localized to the midline anterior skull base, while aggressive tumors showed enrichment in the falcine/parasagittal region and sphenoid wing. Moreover, molecular grading provided sharper spatial clustering than traditional histopathological grading, reinforcing the biological and clinical relevance of voxel-wise analyses.

Building on these foundations, the present study aims to highlight the utility of VLSM for neurosurgical and neuro-oncological research. We apply VLSM to a cohort of 350 patients undergoing surgical resection of intracranial meningiomas, investigating the spatial distribution of histological subtypes, the association with preoperative symptoms and postoperative oncofunctional outcome. We present a methodological extension of VLSM to meningiomas and introduce the dural attachment zone (DAZ) mask as an attachment-focused representation designed to reduce tumor-volume–related spatial spillover compared with total tumor volume (TTV)di masks. We demonstrate this framework across oncofunctional endpoints (motor deficits, extent of resection, recurrence); analyses of histopathological grade serve mainly as an illustrative comparison of TTV- versus DAZ-based mapping. By showcasing these applications, we seek to encourage broader adoption of VLSM methodologies in neurosurgical research and to promote a more standardized, spatially informed approach to studying brain tumors.

## Materials and methods

### Patient population

This retrospective observational single center study enrolled 350 consecutive patients who underwent surgical treatment of intracranial meningioma. The inclusion criteria were defined as sufficient quality of preoperative magnetic resonance imaging (MRI), first-line treatment and complete clinical information about histopathological results. The clinical data was acquired from medical records including age at diagnosis, gender and histopathological findings. In the present methodological study, we focused on the pre- and postoperative presence of motor deficits (MD; i.e., paresis) as we expected a distinct association to a perirolandic meningioma location. Tumor locations were evaluated based on preoperative MRI by experienced, board-certified skull base neurosurgeons. Histopathological diagnosis was reclassified according to the WHO 2021 classification (13). Details of the clinical and demographic characteristics are depicted in **Table 1**. This study was approved by the local ethics committee of the Medical Faculty of the Eberhard Karls University Tuebingen (No. 702/2024B02) and performed in accordance with the Declaration of Helsinki. The results are reported following the STROBE guidelines (14).

**Table 1:** Cohort and tumor characteristics (N = 350).

Variable	Category	Value
Age	Years	56.1 ± 12.8
Sex	Female	262 (75)
	Male	88 (25)
Volume	Mean (ml)	31.6 ± 17.6
Location	Non-skull base	139 (39)
	parasagittal	49 (14)
	falcine	20 (6)
	cerebral convexity	69 (20)
	Skull base	221 (61)
	cavernous sinus	8 (2)
	sphenoid wing*	30 (9)
	clinoidal	34 (10)
	tuberculum sellae	18 (5)
	planum sphenoidale	7 (2)
	olfactory groove	15 (4)
	petroclival	23 (7)
	CPA	34 (10)
	cerebellar convexity	5 (1)
	tentorial	15 (4)
	foramen jugulare	8 (2)
	foramen magnum	7 (2)
	temporobasal	4 (1)
	frontobasal	3 (1)

*medial/lateral sphenoid wing. CPA, cerebellopontine angle.

### Magnetic resonance imaging

Imaging studies were conducted at our institution using 1.5 T MRI scanner (MRI; Siemens Healthineers) including a high-resolution T1-weighted contrast-enhanced MPRAGE sequence (slice thickness: 1.0 mm; TR: 2300 ms; TE: 3.51 ms; TI: 1100; flip angle: 8°; pixel bandwidth: 130; pixel spacing: 1/1 mm; matrix: 256 × 256). Nevertheless, many of the segmentations were carried out on preoperative MRI that have been performed externally, therefore the above listed parameters may vary significantly among patients. The last scan before surgery was used for analyses.

### Voxel-based lesion symptom mapping

Voxel-based lesion–symptom mapping (VLSM) is a voxel-wise statistical approach that relates the anatomical distribution of a lesion to a clinical phenotype. In a common stereotactic space, lesion masks from multiple patients are superimposed, and for each voxel a statistical test compares patients with and without lesion involvement at that voxel with respect to the endpoint of interest. The resulting statistical maps identify brain regions where lesion presence is significantly associated with functional deficits or other clinically relevant outcomes.

**Figure 1:**
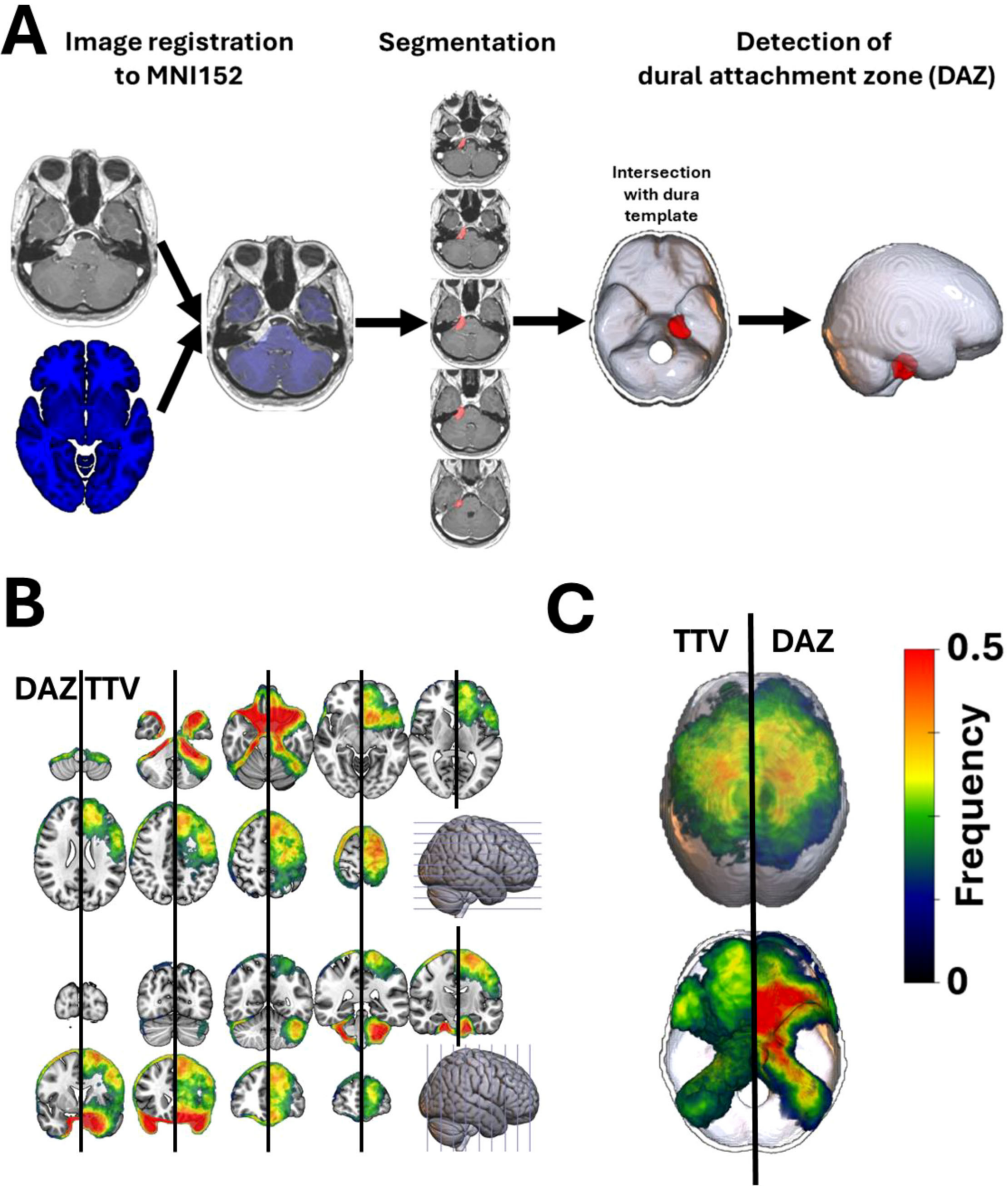
Voxel-based mapping of intracranial meningioma. **(A)** Patients’ individual NIfTI data were registered to a 1-mm isotropic, high-resolution, T1-weighted MRI template provided by the MNI. Total tumor volumes (TTV) were semi-automatically segmented and flipped to the right hemisphere. Tumor masks were intersected with a standardized template representing the dura and falx to detect the dural attachment zone (DAZ) of the meningioma. TTV and DAZ masks were used for the voxel-wise lesion symptom mapping analysis. **(B)** Frequency maps overlayed to an axial and coronal T1 weighted MR template show the spatial distribution of TTV (*left hemisphere*) and DAZ (*right hemisphere*) in the present cohort. Warmer colors indicate higher tumor frequency across the cohort, highlighting anatomical hotspots. Meningiomas were most frequently located at the frontal convexity, the sphenoid wing, and the petroclival junction. In contrast, tumors were rarely observed in the parieto-occipital and cerebellar convexity. **(C)** Frequency maps of TTV and DAZ overlayed to the dural template. Notably, both TTV and DAZ resulted in a similar distribution pattern on the dural level.

All Digital Imaging and Communications in Medicine (DICOM) format images were first converted to the Neuroimaging Informatics Technology Initiative (NIfTI) format by using *dcm2niix* (15). Statistical Parametric Mapping Software version 12 (SPM12, Institute of Neurology, University College London, London, UK; https://www.fil.ion.ucl.ac.uk/spm/docs/) and MATLAB (R2024a, MathWorks, Natick, MA, USA) were used to register and normalize patient’s MR images to a standard brain template (MNI152; Montreal Neurological Institute, McGill University, Montreal, Quebec, Canada) (**Figure 1A**). To reduce potential uncertainty introduced by nonlinear normalization to common stereotactic space, all scans were visually quality-controlled after registration and after mask transformation into MNI space. Segmentations were corrected manually where required, and registration results were checked for gross misalignment of anatomical landmarks and lesion boundaries. In addition, voxel-wise analyses were restricted to voxels involved in at least 10 patients to avoid unstable inference driven by sparse overlap that may be more sensitive to minor normalization deviations. The normalized image was resampled to a voxel size of 1x1x1mm. The tumor was semi-automatically segmented using a fast-marching method implemented in MATLAB (*imsegfmm.m*). The segmentation was performed and reviewed by two neurosurgeons. Manual corrections to the total tumor volume (*TTV*) mask were made in MRIcron (https://www.nitrc.org/projects/mricron/). The individual tumor volume was noted, and the tumor mask was saved for further analysis. All tumor masks were mirrored to the right hemisphere of the brain. Dural attachment zone (*DAZ*) was acquired by intersecting the tumor mask with a template representing the dura, falx and tentorium. This template was generated based on a publicly available high-resolution computer tomography (CT) template (16) and falx/tentorium masks provided by *Brain Biomechanics Imaging Resources Project* (https://www.nitrc.org/projects/bbir). Voxel-based lesion symptom mapping (VLSM) enables to evaluate the relationship between a predictor (e.g., DAZ of the meningioma) and a specific outcome or feature (e.g., histopathological subtype) at individual voxels (i.e., voxel-vice) (17). In this study, VLSM was based on a univariable linear regression model and one-tail t-statistics as implemented in SPM12 (https://www.fil.ion.ucl.ac.uk/spm) and NiiStat (https://www.nitrc.org/projects/niistat). Categorial variables were incorporated after dummy coding (0: reference group; 1: observational group). Only voxels affected in at least 10 patients were included in the VLSM analysis to ensure sufficient statistical power and to minimize the influence of outliers (18). This threshold was chosen to avoid statistically unstable estimates at sparsely sampled voxels (where a small number of cases can drive large apparent effects), to reduce sensitivity to minor segmentation/registration variability, and to ensure that each tested voxel represents a lesion location with sufficient cohort support for meaningful inference. Thus, 193228 voxels were included in the VLSM analysis. VLSM statistics provides Z-scores representing standardized test statistics derived from these models. A positive Z-score indicates a positive association between the occurrence of meningiomas in this voxel and a specific feature (e.g., a specific histopathological subtype). To account for multiple comparisons across the numerous voxels analyzed, we applied three different methods. 1)The Bonferroni method is a wide-spread, easy and fast methods to correct for multiple comparison. However, it is known to be particularly strict (19, 20). 2) False Discovery Rate (FDR) correction controls the expected proportion of false positives among the voxels identified as significant, enhancing the reliability of the results (18, 21). 3) Threshold-free cluster enhancement (TFCE) (22), as implemented by the Threshold-free Cluster Enhancement Toolbox in SPM12 (https://github.com/ChristianGaser/tfce),is known to improve sensitivity and spatial specificity by avoiding arbitrary cluster thresholds. However, it is computationally intensive and relies on tunable parameters (H = 2, E = 0.5) that can influence the results. Multiple comparison correction was based on permutation testing with 5000 permutations. Bonferroni correction was included to provide a family-wise error–controlled reference threshold that is widely reported in the VLSM literature (17), whereas FDR and permutation-based TFCE were additionally applied to illustrate how different established correction strategies influence the spatial extent and localization of significant clusters.

These voxel-wise statistical tests were applied in two complementary contexts: first using the total tumor volume (TTV) masks and then using the dural attachment zone (DAZ) masks. Using the TTV mask in VLSM tests whether tumor presence/expansion at a given voxel is associated with an endpoint. Because larger tumors occupy more voxels, TTV-based inference can be influenced by tumor size and growth-related spatial “spillover” into neighboring regions. In contrast, using the DAZ mask tests whether the part of the tumor anchoring to the dura is associated with the endpoint, thereby providing a more origin-focused anatomical interpretation and reducing volume-driven spatial bias. For example, in the WHO grade analysis, TTV-based mapping showed involvement inside the deep parenchymal structures, whereas DAZ-based mapping highlighted attachment zones along the convexity and frontal base.

### Statistics

Statistical evaluation of clinical data was performed using SPSS (IBM SPSS Statistics for Windows, Version 25.0, Armonk, NY: IBM Corp.) and custom-written MATLAB scripts including MATLAB statistics toolbox. Results are shown as mean ± standard deviation (SD).

### Data availability

The data that support the findings of this study are available from the corresponding author upon reasonable request.

## Results

### Patient cohort and tumor characteristics

The present retrospective study enrolled 350 consecutive patients (age: 56.1 ± 12.8 years) undergoing primary surgical treatment of an intracranial meningioma. Overall, there was a predominance of female sex (approx. 75%). Mean tumor volume was 31.6 ± 17.6 ml. Approx. 61% of meningiomas were located at the skull base. Non-skull base meningiomas were significant larger (45.6 ± 45.7 and 22.5 ± 27.1 ml; H = 6.75; p<0.001) and more likely to present a WHO grade 2 (35% vs 9%; X²=34.31, p<0.001). Meningothelial meningiomas were the most common histological subtype (approx. 49%). Full details are summarized in **Tables 1** and **2**.

**Table 2:** Clinical endpoints and outcomes (N = 350).

Variable	Category	Value
Histology	meningothelial	173 (49)
	fibrous	28 (8)
	transitional	46 (13)
	psammomatous	10 (3)
	angiomatous	10 (3)
	microcystic	8 (2)
	secretory	18 (5)
	lymphoplasmacyte-rich	0 (0)
	metaplastic	3 (1)
	chordoid	8 (2)
	clear cell	0 (0)
	papillary	0 (0)
	rhabdoid	0 (0)
	atypical	44 (13)
	anaplastic	0 (0)
WHO	grade 1	282 (81)
	grade 2	68 (19)
Simpson	Grade 1	93 (27)
	Grade 2	59 (17)
	Grade 3	49 (14)
	Grade>3	67 (19)
	N/A	82 (23)
Motor deficits	preopearive	93 (27)
	postoperative	41 (12)
Follow-up	months	98.2 ± 35.8
Recurrences		69 (20)
Mean-time-to-recurrence		43.1 ± 30.4

### Spatial distribution of intracranial meningiomas

After each individual preoperative MR image was registered to the MNI152 template space, meningiomas were segmented manually. Masks of the total tumor volume (TTV) as well as the dural attachment zones (DAZ) were aggregated to calculate the spatial distribution of all included meningiomas. As illustrated in **Figures 1B, C**, TTV-based VLSM and DAZ-based VLSM address different anatomical questions (expansion vs attachment). For WHO grade mapping, TTV-VLSM produced intracranial clusters extending in the brain parenchyma, whereas DAZ-VLSM localized associations to attachment regions, yielding a more interpretable origin-focused pattern. The resulting frequency maps revealed meningioma hotspots in the anterior two-third of the falx cerebri, the frontal convexity, and skull base structures such as the sphenoid wing and petroclival junction. In contrast, only a few lesions were observed in the parieto-occipital convexities. Notably, both TTV and DAZ resulted in a similar distribution pattern on the dural level. However, TTV masks also depict intracranial meningioma extension.

### Tumor volume bias in voxel-based lesion symptom mapping

**Figure 2:**
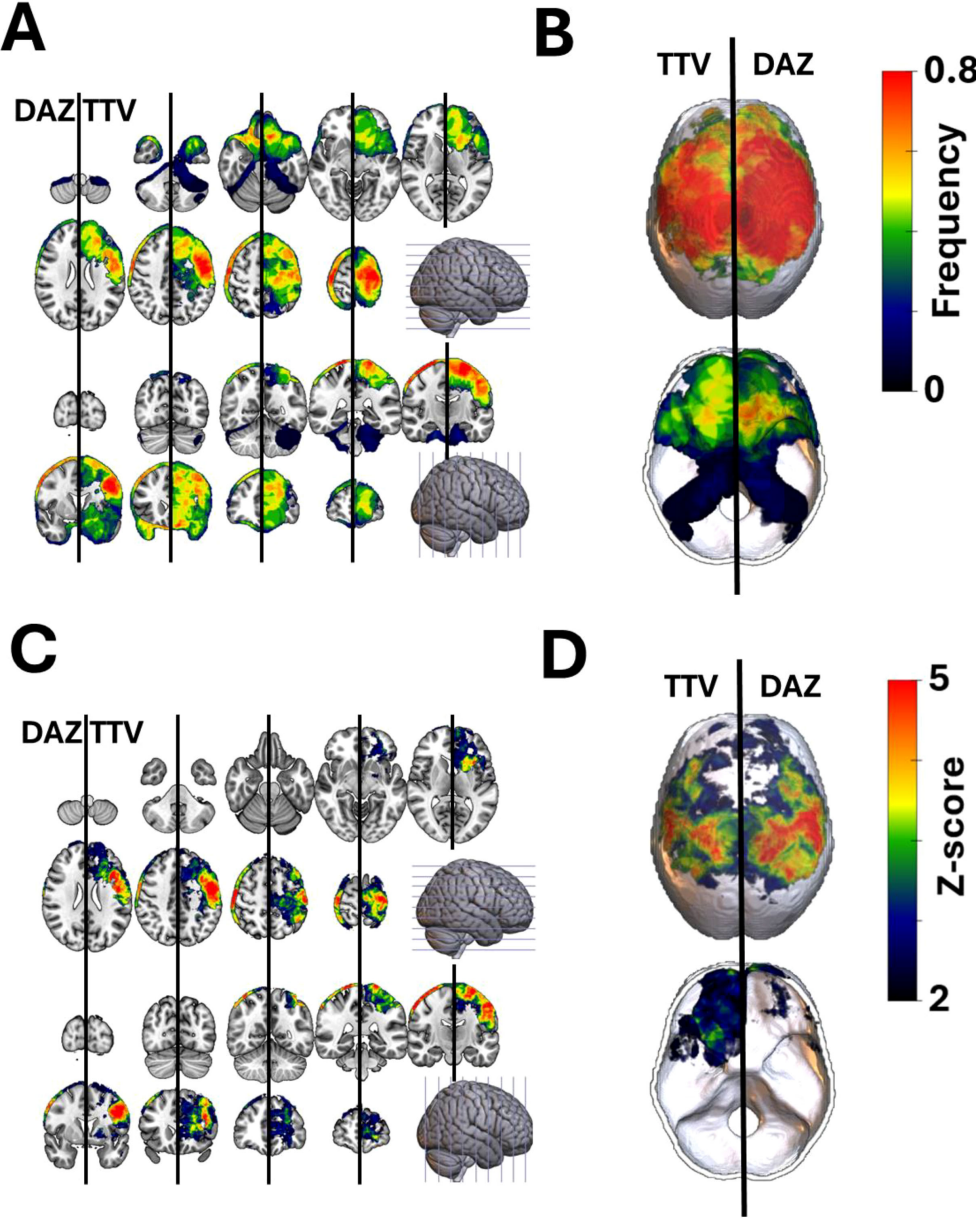
Spatial distribution of WHO grade 2 meningiomas. Frequency maps of total tumor volumes (TTV) and dural attachment zones (DAZ) of WHO grade 2 meningiomas overlayed on a standardized T1-weighted MR **(A)** and dural template **(B)**. Voxel-based lesion symptom mapping (VLSM) applied on TTV and DAZ masks indicated a significant predominance of WHO grade 2 meningiomas on the cerebral convexity and frontal base. Significant Z-scores are thresholded and corrected for multiple comparisons (p<0.05; FDR) **(C, D)**.

Voxel-based lesion–symptom mapping (VLSM) was performed using both total tumor volume (TTV) and dural attachment zone (DAZ) masks to identify statistically significant clustering of WHO grade 2 meningiomas (**Figure 2**). This side-by-side comparison highlights that DAZ-based mapping primarily improves interpretability by reducing volume-driven extension of significant clusters beyond plausible dural origin regions. In fact, WHO grade 2 lesions clustered along the parasagittal cerebral convexity and the frontal skull base. However, TTV-based VLSM also revealed prominent frontal‐lobe clusters that did not correspond to any distinct dural attachment zone. Because non–skull-base meningiomas are on average larger than their skull-base counterparts, the overlap between frequency and significance maps in TTV-based VLSM suggests that volume, rather than true anatomical predilection, drives these findings. By focusing analysis on the DAZ and effectively normalizing for overall tumor size, DAZ-based VLSM attenuates this volume bias and enables more anatomically precise identification of lesion–outcome relationships.

### Effect of different multiple comparison corrections

**Figure 3:**
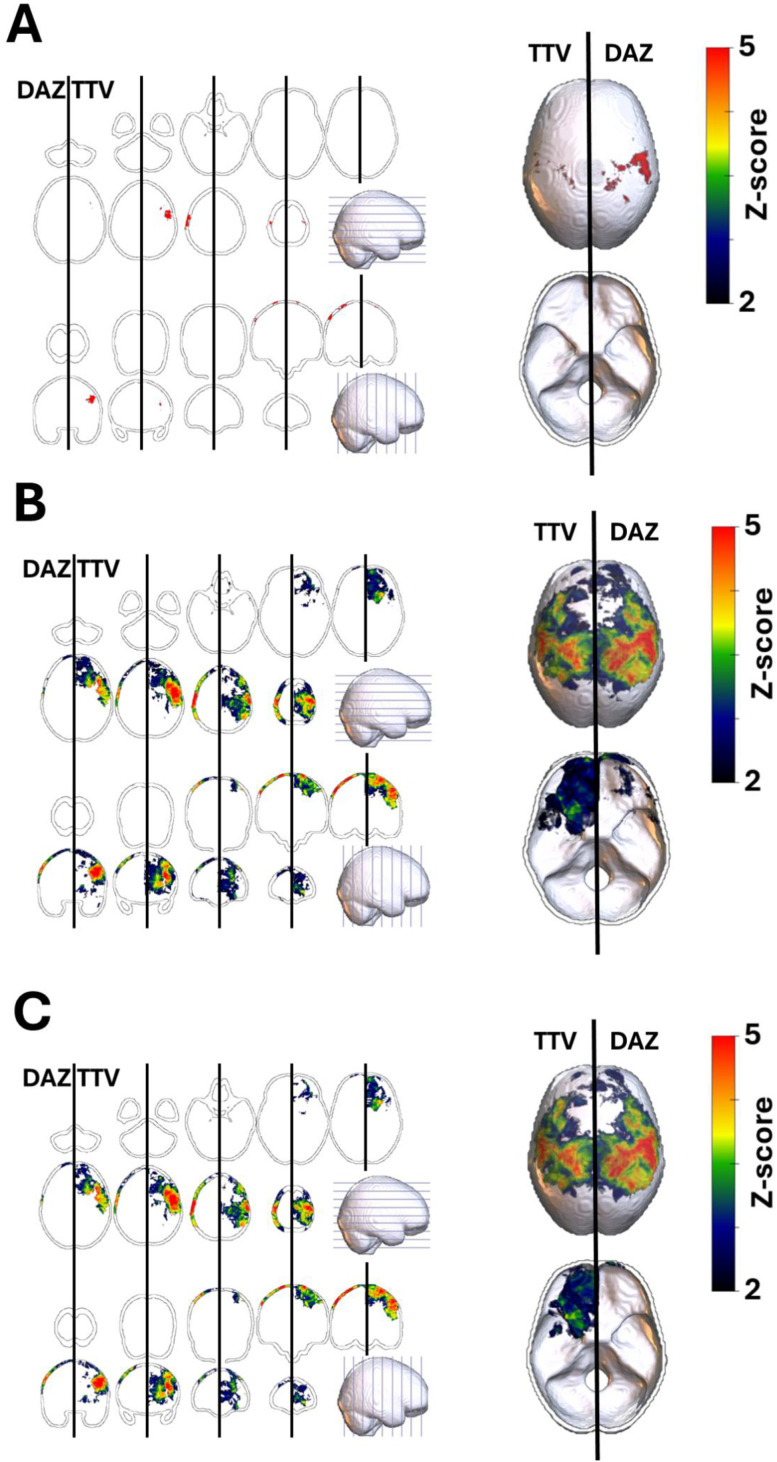
Effect of different multiple comparison corrections. Voxel-based lesion symptom mapping (VLSM) results for the spatial distribution of WHO grade 2 meningiomas, corrected using three different methods for multiple comparisons: **(A)** Bonferroni correction (BON), **(B)** false discovery rate (FDR), and **(C)** threshold-free cluster enhancement (TFCE). The Bonferroni correction was highly conservative, resulting in few significant clusters. FDR and TFCE produced comparable overall patterns; however, TFCE tended to suppress smaller clusters (e.g., at the frontal skull base).

The issue of multiple comparisons is an inherent methodological limitation in VLSM studies. In the present study, we evaluated the effect of three different methods for multiple comparison correction on the VLSM results on WHO grade 2 meningioma (**Figure 3**). The Bonferroni correction was highly conservative, resulting in few significant clusters. FDR and TFCE produced comparable overall patterns; however, TFCE tended to suppress smaller clusters (e.g., at the frontal skull base).

### Spatial relationship between meningioma location and symptomatology, surgical resectibility and oncofunctional outcome

**Figure 4:**
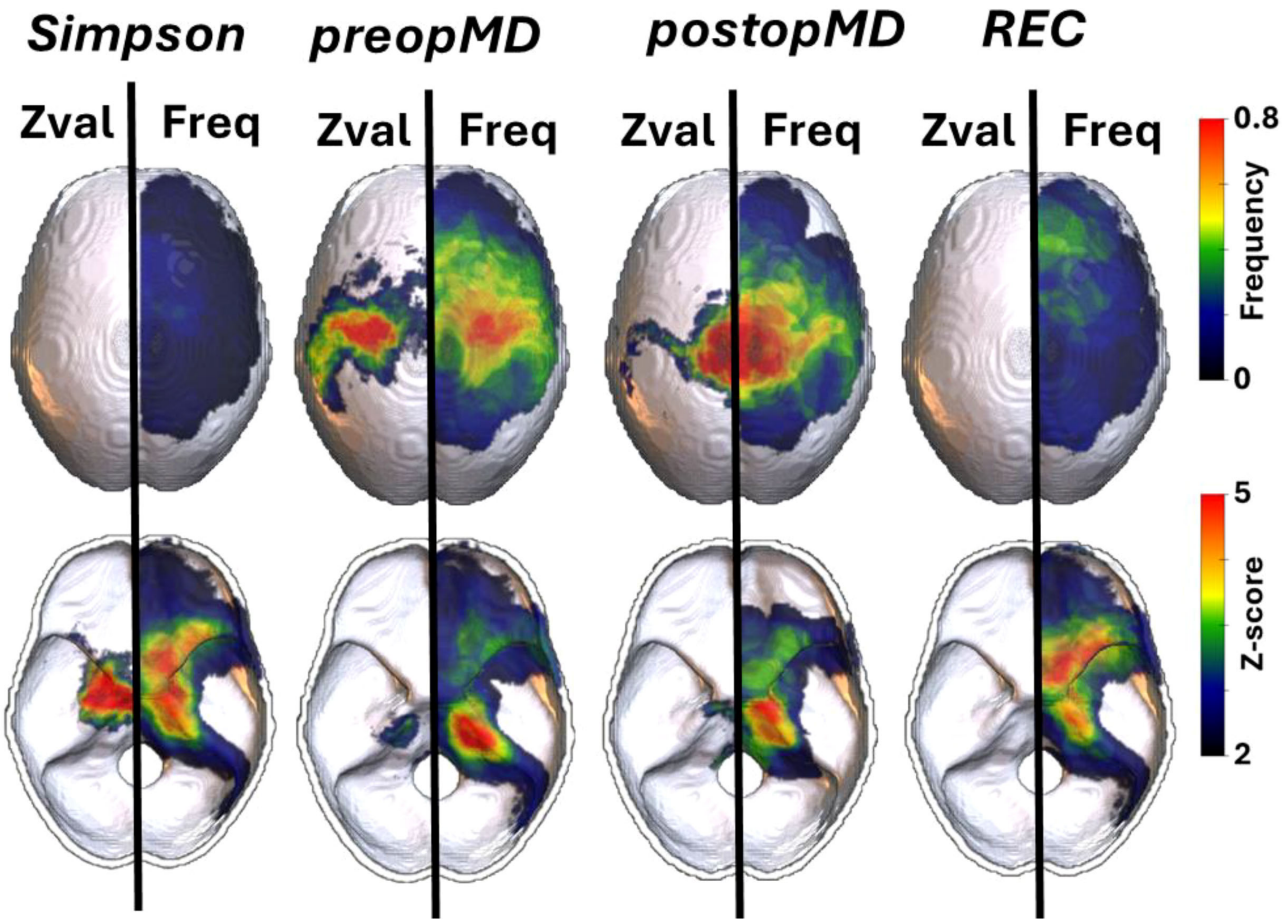
Spatial distribution of meningioma DAZ depending on surgical performance (i.e., SIMPSON grading), pre- and postoperative functional outcome. Frequency maps (Freq) and VLSM results (Zval) projected on the left and right hemisphere. Meningiomas of the medial sphenoid wing correlated with a higher risk for subtotal resection (Simpson grade >3). Meningiomas associated with preoperative (preopMD) and postoperative motor deficits (postopMD) showed significant clustering at the perirolandic and petroclival area. We could not detect any significant spatial predominance of recurrent meningiomas (REC) in the present study. Significant Z-scores are thresholded and corrected for multiple comparisons (p<0.05; FDR).

Preoperative motor deficits (preopMD) were observed in 27%. VLSM was performed to identify statistically significant clustering of meningioma DAZ associated with preopMD. Here, we observed a significant clustering at the perirolandic and petroclival area (**Figure 4**). In 27% of case no total resection was achieved (Simpson grading >3). VLSM identified a significant association of medial sphenoid wing meningioma with preop MD (**Figure 4**). When applying VLSM on oncofunctional outcome measures (i.e., postoperative motor deficits and the occurrence of recurrences). While postoperative motor deficits (postopMD) showed significant clustering at the perirolandic and petroclival area, there was no significant spatial predominance of recurrent meningiomas in the present study (**Figure 4**).

## Discussion

Voxel-based lesion symptom mapping (VLSM) offers a powerful and statistically rigorous approach for relating lesion location to clinical outcomes. Although VLSM was originally developed in the context of neurological disorders such as stroke (1, 2), its application to tumor cohorts, particularly meningiomas, presents unique methodological opportunities and challenges. This work primarily contributes a methodological framework for applying VLSM to extra-axial tumors and proposes DAZ-based mapping as an attachment-focused complement to conventional whole-tumor (TTV) mapping. Across clinically relevant oncofunctional endpoints, DAZ-based maps yield anatomically interpretable localization tied to dural attachment regions, consistent with the conceptual goal of reducing volume-driven spatial spillover inherent to whole-tumor masks (**Table 3**). While some spatial patterns observed for histopathological grade resemble prior reports, these results are presented here chiefly to demonstrate how inference and interpretability can differ between TTV- and DAZ-based analyses within the same voxel-wise statistical framework.

**Table 3:** Summary of significant VLSM findings by endpoint.

Endpoint	Significant clusters
WHO grade 2 meningiomas	cerebral convexity and frontal skull base.
Preoperative motor deficits	perirolandic area and petroclival area
Postoperative motor deficits	perirolandic area and petroclival area
Subtotal resection	medial sphenoid wing
Recurrence	No significant spatial predominance

Our VLSM results on meningiomas mostly confirm known findings from literature. As there is currently no standardized anatomical classification of meningioma distribution across the cranial vault, comparisons between different studies remain challenging. One of the most comprehensive recent descriptions of meningioma locations was provided by Hosainey et al. (23) in a cohort of 602 patients, reporting a spatial distribution mirroring the findings of the present study. Their data showed that meningiomas were most commonly located at the cerebral convexity (25%), followed by the parasagittal region (16%), sphenoid wing and clinoid (16%), olfactory groove (8%) and falx (8%) (23). However, frameworks such as that of Hosainey et al.

inevitably impose arbitrary spatial boundaries and cannot capture the continuous topography of tumor-clinical associations. By contrast, VLSM obviates the need for predefined regions of interest (ROIs): it evaluates each voxel in a common brain space for its statistical relationship to histology or functional outcome. This voxel-level approach both minimizes bias introduced by region size and boundary definition, and enhancing anatomical specificity and facilitating direct meta-analysis across cohorts. A key factor of voxel-wise mapping approaches is therefore nonlinear normalization, which introduces potential spatial uncertainty, particularly in the presence of mass effect or variable imaging protocols. Importantly, because DAZ-based mapping emphasizes attachment regions (dural compartments) rather than intraparenchymal voxel boundaries, it may be less sensitive to small registration deviations than whole-tumor expansion patterns.

To date, only three studies have applied voxel-based spatial analyses to describe the anatomical distribution of intracranial meningiomas (10–12). Despite methodological differences, they consistently identify predilection sites along the midline parasagittal region, with a hotspot at the anterior two-thirds of the superior sagittal sinus and falx, the frontobasal region, and the cerebellopontine angle (10–12). In our and in other cohorts, WHO grade 2 meningiomas represented approx. 20% of the cases (24). We found a significant spatial clustering of WHO grade 2 meningiomas at the cerebral convexity. This finding is in line with previous research indicating a higher incidence of WHO-grade 2 meningiomas in non-skull base locations (11, 12, 25, 26). In evaluating functional outcomes, VLSM provides an unbiased, whole-brain analysis of how tumor location affects postoperative neurological deficits. Expectedly, meningiomas of the perirolandic region were associated with a significant higher risk of postoperative motor deterioration (27, 28). By identifying regions where tumors are associated with greater risks of neurological symptoms and postoperative impairment, VLSM can inform both patient counseling, preoperative risk stratification, surgical planning and intraoperative decision-making. Moreover, because meningiomas generally cause reversible compression rather than irreversible tissue destruction, VLSM analyses in these patients may capture both the risk of transient postoperative deficits and the potential for functional recovery following tumor resection.

Meningiomas differ from other brain lesions in important ways. Whereas strokes and intra-axial tumors (e.g., gliomas) typically destroy brain tissue, meningiomas exert mass effect by displacing and compressing adjacent brain structures. Their discrete anatomical boundaries facilitate precise lesion segmentation, minimizing errors during image processing (7, 29). The superficial displacement caused by meningiomas usually spares deep brain structures, which facilitates more accurate non-linear normalization to standard brain templates (8). In contrast, normalization can be hindered in lesions with massive affection of brain anatomy such as stroke, intra-axial tumors (e.g., gliomas) or massive brain edema (8).

In this study, we deliberately focused our VLSM analysis on the dural attachment zone (DAZ) rather than on the total tumor volume (TTV). This approach offers several advantages. First, the dural attachment represents the site of tumor origin and biological anchorage, providing a more anatomically and biologically relevant basis for spatial analyses. Second, using the DAZ reduces the potential for volume-related bias, a well-recognized limitation in VLSM studies. Larger tumors, by their nature, occupy more voxels and may artificially inflate associations with certain regions, thereby distorting spatial findings (30, 31). Additionally, larger lesions tend to cause more severe deficits regardless of location (32). By restricting analyses to the DAZ, we minimize this confounding factor, achieve a more accurate mapping of spatial predilection patterns and facilitate statistical analysis. Several methodological strategies have been proposed to address volume bias, including statistical correction for lesion size, matched subgroup analyses, or voxel inclusion thresholds (30, 31). In meningiomas, however, the anatomical specificity provided by focusing on the DAZ remains a particularly effective and biologically meaningful solution. So far, none of the VLSM studies on meningiomas have applied the concept of DAZ in their analysis (10–12).

Normalization of imaging data to a common stereotactic space not only improves the anatomical comparability within a given cohort but also enables direct comparison across independent studies. This feature represents a major strength of VLSM, as it allows researchers to pool spatial data, apply uniform analytical pipelines, and benchmark new findings against established lesion maps or atlases. This evident spatial predominance of driver mutations leads to the development of radiogenomic “mutation maps” (33). Since then, these maps have been reproduced and refined in subsequent studies (34–40). Comparing the distribution pattern of meningiomas with maps radiogenomic mutation maps alterations could yield powerful multidimensional models of tumor behavior. The work of Patel et al. provides a compelling example of how voxel-wise analyses can be combined with molecular data to elucidate phenotype-genotype relationships (12). Furthermore, longitudinal VLSM analyses could offer new insights into how tumor growth patterns or recurrence are related to functional outcomes over time. By anchoring tumor localization within a common coordinate system, spatial analyses become replicable, integrative, and extensible beyond individual institutional datasets, thus fostering multi-center collaborations and meta-analytic efforts.

Nevertheless, some challenges must be acknowledged. Variability in tumor size, the extent of peritumoral edema, and postoperative changes can introduce noise into lesion maps. Rigorous statistical approaches, including corrections for multiple comparisons, are essential to ensure the validity of results (19, 20). Additionally, while VLSM excels at detecting focal associations between lesions and outcomes, it may miss network-level dysfunction, which is increasingly recognized as critical in understanding brain-tumor interactions. Complementary approaches, such as lesion-network mapping, should be considered in future research to provide a more holistic view of brain-tumor dynamics (41).

## Conclusion

In conclusion, voxel-based lesion symptom mapping holds substantial promise for advancing neurosurgical and neuro-oncological research. VLSM can uncover critical spatial patterns associated with tumor histology, molecular alterations, functional deficits, and oncological outcomes. The use of spatial normalization to a common brain space not only strengthens internal study validity but also enhances external comparability, facilitating pooled analyses and fostering collaborative efforts in neuro-oncology research. We advocate for the broader adoption of VLSM methodologies in neurosurgical research and clinical practice to facilitate more precise, individualized, and scientifically grounded approaches to patient care.

## Data Availability

The raw data supporting the conclusions of this article will be made available by the authors, without undue reservation.
